# Computational Microscopy Reveals Compound-Specific Flickering Phenotypes of Red Blood Cells Under Flavonoid Exposure

**DOI:** 10.3390/membranes16030095

**Published:** 2026-03-03

**Authors:** Carlos del Pozo-Rojas, Sandra Montalvo-Quirós, Lourdes Rufo, José María Bueno, Macarena Calero, Francisco Monroy, Diego Herráez-Aguilar

**Affiliations:** 1 Instituto de Investigaciones Biosanitarias, Facultad de Ciencias Experimentales, Universidad Francisco de Vitoria, Ctra Pozuelo-Majadahonda km 1.800, 28223 Madrid, Spain; carlos.delpozo@ufv.es (C.d.P.-R.); sandra.montalvo@ufv.es (S.M.-Q.); l.rufo.prof@ufv.es (L.R.); jmaria.bueno@ufv.es (J.M.B.); 2 Departamento de Química Física, Facultad de Ciencias Químicas, Universidad Complutense de Madrid, Av. Complutense s/n, 28240 Madrid, Spain; macacale@ucm.es; 3 Translational Biophysics, Instituto de Investigación Sanitaria Hospital Doce de Octubre (IMAS12), Av. Andalucía, 28041 Madrid, Spain; 4 Faculty of Health Sciences-HM Hospitals, Camilo Jose Cela University, Villanueva de la Cañada, 28692 Madrid, Spain; 5 HM Hospitals Health Research Institute, 28015 Madrid, Spain

**Keywords:** erythrocytes, membrane mechanics, flickering, flavonoids, quercetin, rutin, apigenin, computational microscopy, biophysics

## Abstract

Red blood cell (RBC) membrane flickering arises from the interplay between thermal fluctuations, cytoskeletal elasticity, and metabolically driven non-equilibrium processes, making it a sensitive reporter of membrane mechanical state. Here, we introduce a computational microscopy framework that integrates bright-field morphometry with high-speed flickering spectroscopy to phenotype single-cell RBC mechanics under flavonoid exposure. As a proof of concept, human erythrocytes from a single donor were incubated with structurally distinct flavonoids (quercetin, apigenin, and rutin) prepared at sub-hemolytic concentrations, ensuring preservation of membrane integrity. Static shape descriptors and dynamic fluctuation spectra were extracted from segmented cell contours and analyzed through Fourier-mode decomposition to obtain compound-specific mechanical signatures. While gross morphology remained largely discocytic across conditions, flavonoid treatment induced reproducible alterations in flickering spectra and effective mechanical parameters, revealing distinct dynamical phenotypes that depend on flavonoid structure. In particular, aglycone flavonoids exhibited modulation patterns that differed from the glycosylated compound, consistent with differential membrane interactions. The combined analysis of geometry and dynamics provided enhanced discriminative power compared to either modality alone. These results establish computational microscopy as a sensitive, label-free approach to map compound-specific perturbations of RBC membrane mechanics and flickering, with potential applications in membrane biophysics, drug–membrane interaction screening, and single-cell mechanical phenotyping.

## 1. Introduction

Red blood cells (RBCs) are an archetypal system for linking molecular-scale membrane organization to cell-scale mechanics and function [1]. Their ability to repeatedly deform while traversing microcapillaries depends on a composite envelope: a lipid bilayer coupled to an underlying spectrin–actin cytoskeleton through transmembrane complexes [2]. Perturbations to either the bilayer (e.g., lipid composition, cholesterol content) or the membrane skeleton (e.g., connectivity, anchoring) can shift RBC shape, deformability, and susceptibility to hemolysis, with direct physiological relevance [3,4]. Beyond bulk rheology, RBCs are particularly attractive because key mechanical and functional readouts (shape, membrane fluctuations, and effective mechanical parameters) are accessible at the single-cell level using optical microscopy and quantitative analysis pipelines.

At rest, healthy RBCs exhibit a stable biconcave morphology and a characteristic spectrum of nanometer-scale membrane fluctuations, aka “flickering” [5]. Flickering arises from the interplay between thermal forces, bilayer bending resistance, membrane tension, and cytoskeletal shear elasticity, and it can be further modulated by non-equilibrium, metabolism-dependent activity [6,7,8]. Because flickering integrates multiple structural and energetic contributors, it provides a compact, systems-level mechanical phenotype that is sensitive to perturbations affecting bilayer–cytoskeleton coupling, membrane viscosity, or active processes [9]. In this sense, simultaneously characterizing RBC shape (morphometry) and flickering (dynamics) offers a coherent framework to track how a chemical perturbation propagates across scales, from molecular interactions at the membrane to observable cellular phenotypes.

Multiple approaches have been developed to quantify RBC flickering and infer effective mechanical parameters. Eigenmode-based decompositions and spatially resolved fluctuation analyses can extract mode amplitudes and assess the contribution of ATP-dependent processes [6,10], while correlation-based strategies quantify spatiotemporal correlations, often using quantitative phase imaging, to connect fluctuation statistics with effective mechanical descriptors through model-based inference under controlled perturbations [8,11,12,13]. Although differing in implementation, these methods converge on a shared premise: fluctuation spectra and their spatial structure encode the effective mechanics of the RBC envelope. For comparative studies across compounds, this motivates adopting a homogeneous image-processing and analysis pipeline to minimize methodological variance and to enable robust, side-by-side mechanical phenotyping.

**Figure 1:**
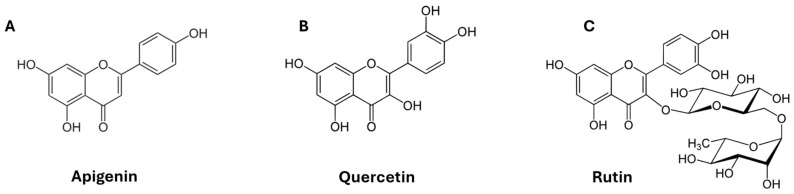
Chemical structures of the flavonoids investigated: (**A**) apigenin, (**B**) quercetin, and (**C**) rutin. The figure highlights the structural differences between the aglycones (apigenin and quercetin) and the glycosylated compound (rutin), which are discussed in relation to their membrane affinity and compound-specific flickering phenotypes.

Flavonoids are a chemically diverse family of plant-derived polyphenols widely studied for antioxidant and other bioactivities, and they also represent prototypical small molecules whose structure strongly governs membrane affinity, depth of insertion, and perturbation of lipid order. Mechanistic studies emphasize that flavonoid–membrane interactions depend on hydroxylation pattern, molecular planarity and conjugation, and the presence of sugar moieties, which increase polarity and typically reduce bilayer partitioning [14,15,16]. In model membranes, quercetin has been shown to interact with lipid bilayers and modify membrane properties, and comparative work suggests substantial variation in membrane affinity and effects across flavonoids [17,18]. These considerations motivate focusing here on three representative compounds: quercetin (a flavonol aglycone), rutin (a glycosylated quercetin derivative), and apigenin (a flavone aglycone) (see chemical structures in Figure 1).

In erythrocytes, much of the flavonoid literature has historically emphasized protection against oxidative stress and membrane-damage endpoints (hemolysis, lipid peroxidation, protein oxidation), often under exogenous oxidant challenge. Quercetin has long been reported to protect erythrocyte membranes against oxidative damage, with mechanistic interpretations including iron chelation and attenuation of lipid peroxidation [19], and other studies have reported measurable changes in membrane organization and, in some cases, shape alterations [20]. A recurring theme is that membrane composition, especially cholesterol, modulates flavonoid effects: cholesterol regulates bilayer order and lateral organization, influences small-molecule partitioning [21], and affects the mechanical coupling between bilayer and cytoskeleton [22]. In oxidatively stressed erythrocytes, cholesterol has been reported to modify the protective effects of quercetin and rutin on integrity and viability, underscoring the importance of membrane context and reinforcing that rutin (as a polar glycoside) cannot be assumed to behave as a simple equivalent of quercetin at the membrane interface [23,24]. More broadly, RBC mechanics and flickering are strongly responsive to cholesterol-dependent organization [9] and to amphiphilic membrane active agents such as beta escin, which remodel bilayer mechanics [25,26] and bilayer cytoskeleton coupling through mechanisms distinct from polyphenolic insertion. Although beta escin is chemically distinct from flavonoids, being a triterpenoid saponin rather than a polyphenol, it shares an amphiphilic architecture characterized by a membrane-active aglycone core and glycosidic substituents that modulate polarity and membrane partitioning. The characteristic mechanical impact of escin on RBC flickering highlights how chemically distinct phytochemical classes can generate distinct and interpretable membrane phenotypes [27]. Apigenin adds a further mechanistic dimension because its scaffold and hydroxylation pattern differ from quercetin; beyond model-membrane studies [20], apigenin has been linked to erythrocyte-specific responses such as eryptosis-like hallmarks [28] and has been reported to reduce hemolysis and oxidative markers in oxidative-stress models [29]. Apigenin therefore appears to act in a regime-dependent manner rather than being uniformly protective or deleterious, with outcomes strongly shaped by dose, incubation time, and the oxidative context [28,30].

Collectively, these reports motivate treating quercetin, rutin, and apigenin as mechanistically distinguishable perturbations with potentially distinct consequences for whole-cell mechanics and dynamics (see Table 1).

Despite this substantial biochemical and membrane-focused literature, a practical gap remains from the perspective of membrane biophysics and single-cell phenotyping: comparatively few studies provide a systematic, side-by-side quantification of both RBC morphometry and membrane flickering across multiple flavonoids using a consistent experimental and analytical workflow. This dual approach matters because morphology and fluctuations are not redundant: a compound may remodel bilayer–skeleton coupling and shift fluctuation spectra without producing an obvious mean-shape transition or, conversely, induce shape alterations with modest changes in fluctuation statistics. Accordingly, the objective of this work is to evaluate whether quercetin, rutin, and apigenin generate reproducible, distinguishable signatures in RBC morphometry and membrane flickering under matched conditions using a homogeneous computational microscopy pipeline. The working hypothesis is intentionally conservative: given their structural differences and known determinants of membrane interaction [14,15], these flavonoids will not produce equivalent perturbations at the RBC membrane, and this non-equivalence will be observable as compound-specific shifts in single-cell shape descriptors and/or fluctuation-derived metrics. A secondary hypothesis is that combining morphometry with flickering yields higher discrimination power than either readout alone because it jointly samples static geometry and dynamical mechanical behavior of the same composite membrane system.

Importantly, the present study is framed as a proof-of-concept at the phenotype level. Our primary aim is to establish feasibility and validate the end-to-end computational microscopy pipeline under controlled, sub-hemolytic conditions, rather than making population-level claims. This framing also defines the work as a methodological framework to support subsequent expansion to multi-donor cohorts, dose–response designs, and cholesterol-modulation experiments.

## 2. Materials and Methods

### 2.1. Reagents and Solutions

Apigenin, quercetin, and rutin (≥95% purity) were used as membrane-active effectors. Apigenin (CAS 520-36-5) and quercetin (CAS 117-39-5) were obtained from Merck Life Science S.L.U (Madrid, Spain). Rutin (CAS 153-18-4) was purchased from Phyto-Lab GmbH & Co. KG (Vestenbergsgreuth, Germany). Stock solutions were prepared in DMSO (100%) and diluted in incubation buffer to the desired working concentrations. DMSO content was kept constant across all groups, including the vehicle control (final DMSO 0.1% *v*/*v*).

A PBS-based incubation medium enriched with glucose and albumin (PBS+) was prepared using 1× PBS (pH 7.4) supplemented with D-glucose (1.8 mg/mL) and bovine serum albumin (BSA; 1.0 mg/mL). Solutions were filtered (0.22 µm) and stored at 4 °C until use.

### 2.2. Blood Collection and Erythrocyte Preparation

Fresh blood was obtained by capillary finger-prick from a healthy adult donor. Whole blood was diluted 1:10 in PBS+. The suspension was centrifuged (5000× *g*, 10 min, 4 °C), the supernatant discarded, and the erythrocyte pellet resuspended in PBS+. The washing step was repeated, and the final washed erythrocyte suspension was adjusted to a final volume of 500 µL. Samples were maintained at 37 °C and used within 3 h of preparation.

### 2.3. Flavonoid Stock Solutions and Working Concentrations

Flavonoids were dissolved in 100% DMSO to obtain concentrated stock solutions and subsequently diluted in PBS+ to yield the desired working concentrations while maintaining a constant final DMSO content of 0.1% (*v*/*v*) across all experimental conditions. For long-term cytotoxicity assays and for imaging experiments (morphometry and flickering), the following concentrations were selected: apigenin 10 µM, quercetin 50 µM, and rutin 50 µM. All flavonoid solutions were prepared fresh on the day of each experiment and handled under light-protected conditions to minimize degradation of photosensitive compounds.

### 2.4. Experimental Design and Definition of Replicates

Experimental groups included a baseline vehicle control (PBS+ + 0.1% DMSO) and three flavonoid-treated groups: apigenin, quercetin, rutin (in PBS+ + 0.1% DMSO). Vehicle controls were evaluated at two time points (C0h and C1h) and the corresponding flavonoid-treated conditions after 1 h of incubation. The baseline control (C0h) corresponds to vehicle-treated erythrocytes analyzed immediately after extraction and purification and therefore reflects a transient post-isolation state, as the extraction and washing procedures impose acute mechanical and osmotic stress on the cells. The incubated control (C1h) corresponds to vehicle-treated erythrocytes after 1 h of incubation at 37 °C, allowing partial recovery from extraction-induced stress. All morphometric and flickering measurements in flavonoid-treated samples were performed after 1 h of incubation at 37 °C and are therefore directly comparable to the C1h vehicle control. For the integrity assessment, hemolysis was quantified in control samples at 30, 60, 90, 120, and 180 min, whereas flavonoid-treated samples were evaluated at 30, 90, and 180 min. To ensure statistical significance, the experiments were carried out in triplicate.

### 2.5. Hemolysis Assay (Integrity Control)

To quantify hemolysis, samples were centrifuged after the selected incubation period (5000× *g*, 10 min, 4 °C), and the supernatant was collected for hemoglobin determination. Hemoglobin absorbance was measured by scanning spectrophotometry (Thermo Scientific™ GENESYS™ 30, Madison, WI, USA) using the Harboe approach, i.e., the primary readout at 415 nm with baseline corrections at 380 nm and 450 nm [33]. For blank correction, two condition-matched blanks were prepared and measured in parallel: (i) PBS + 0.1% DMSO (vehicle control) and (ii) PBS + 0.1% DMSO + flavonoid (treatment vehicle), to account for the optical contribution of solvents and compounds. Blank optical densities were subtracted from the measured absorbance at each wavelength prior to applying the Harboe equation to obtain hemoglobin concentration (Appendix B.1) [15,34,35,36].

Hemolysis is reported as a percentage relative to complete hypotonic lysis in deionized water (100% hemolysis). All conditions (vehicle control and flavonoid-treated samples) were assayed in triplicate. The limit of detection (LOD) was estimated separately for each condition from replicate blank measurements: three independent blank replicates (vehicle control and each flavonoid vehicle) were prepared, and each replicate was measured five times to quantify the absorbance uncertainty used for LOD determination.

### 2.6. High-Throughput Morphmechanical Phenotyping

Aliquots (40 µL) were diluted 2:15 in PBS+ and loaded into 8-well chamber slides. Bright-field images were acquired on a Leica THUNDER inverted microscope (Leica Microsystems GmbH/CMS GmbH, Wetzlar, Germany) using a Leica K5 camera (LAS X) and an HC PL APO 63×/1.4 oil objective (0.103 μm/px). Images were recorded at 2048 × 2048 px. Ten fields per sample at 60 min were used for quantitative analysis, at a constant temperature of 37 °C, with a typical amount of 30–40 cells per field (300–400 cells per sample).

Image processing for morphometric analysis was performed in Wolfram Mathematica version 14.3 (Wolfram Research, Inc., Champaign, IL, USA) using custom-written scripts (see Code Availability Statement). Bright-field images were first subjected to noise reduction by Gaussian filtering (radius 2 px), globally normalized and subsequently binarized using a global threshold determined by Otsu’s clustering-based variance maximization method. Connected components were then labeled to identify candidate erythrocytes. Components were accepted for further analysis only if they corresponded to isolated cells fully contained within the field of view and in focus; aggregates, border-touching objects, and out-of-focus cells were excluded. In addition, quantitative acceptance criteria were applied to restrict the analysis to suspended normocytic erythrocytes displaying minimal morphological alterations. Specifically, components were required to exhibit a circularity greater than 0.9, to exclude elliptocytes and echinocytes, and an equivalent radius between 7 and 8 µm, to avoid stomatocytes, reticulocytes, or abnormally sized cells. Under these criteria, approximately 10% of the initially detected components were typically discarded.

**Figure 2:**
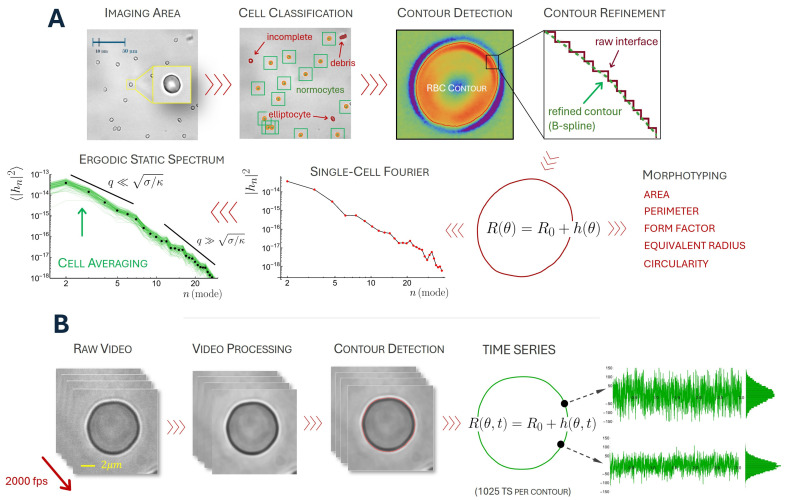
Processing of cell field images and single-cell videos. (**A**) Processing of cell field images and extraction of shape properties from the selected components. Each image was analyzed to detect high-contrast regions corresponding to cell membranes and subsequently binarized to extract individual component shapes. Circularity, elongation, and area criteria were applied to discard non-usable components (non-normocytes). Cell contours were obtained and used as membrane interfaces after applying a 12th-degree B-spline polynomial interpolation. (**B**) Extraction of membrane fluctuation time series. Each single-cell video was analyzed and processed, and an 18th-degree B-spline polynomial interpolation was applied to extract 1024 membrane interface points for each of the 6000 frames, generating 6000 × 1025 time series per video. Representative time series for two membrane points are presented.

Accepted cell contours were extracted using Otsu-based segmentation and further refined to mitigate pixelation effects (Figure 2A). The raw contours were transformed into polar coordinates interpolated using a B-spline polynomial of degree twelve and resampled on a fixed angular grid of 720 points spanning 0 to 2π. This procedure ensured smooth, uniformly sampled contours suitable for robust and reproducible computation of morphometric descriptors. Cell contours were described as parametric curves, $R\left(\theta \right)=R_{0}+h(\theta )$, shaped as a mean radius $R_{0}$ (or equivalent disk radius) with an angular-dependent perturbation h(θ). For each accepted cell, we computed the following morphological parameters: area (A), perimeter (P), circularity ($C=2\;\pi \;R_{0}/P$), form factor ($F=P^{2}/A$), and elongation (E=a/b), where a and b are the major and minor semi-axes of the inertia-equivalent ellipse fitted to the cell contour.

### 2.7. Ergodic Approximation

In addition to morphological estimations, we have performed Helfrich-like physical analysis of membrane fluctuations by evaluating $h\left(\theta \right)$ for each cell at a given time-shot in an ergodic fashion, following refs. [37,38]. The erythrocyte population was assumed to be biologically homogeneous and to behave as an ergodic system, in such a way that temporal averaging of membrane fluctuations for a given cell is equivalent to ensemble averaging over the population, which exhibits morphological variability at any given time. This approach allows for a higher-throughput comparative characterization of the mechanical properties of the sample. The validity of this ergodic approximation for estimating effective membrane tension has been previously assessed by direct comparison with conventional time-resolved flickering analyses under matched conditions [9,13]; in the present proof-of-concept study, we use it strictly as a comparative estimator under a fixed acquisition/analysis pipeline. Effective tensions inferred from ensemble-averaged static spectra have been shown to quantitatively agree, within experimental uncertainty, with tensions obtained from temporal fluctuation spectroscopy on individual cells under matched conditions [9,13]. This cross-validation supports the use of ensemble-based static spectra as a reliable estimator of effective lateral tension for comparative studies across conditions. The ergodic method is implemented as follows:

First, the mean fluctuation amplitude for each cell was computed as the standard deviation of the contour fluctuations, defined as $\Delta_{i}={\left({\left\langle h^{2}\right\rangle }_{\theta }\right)}^{1/2}$, where ⟨·⟩ represents averaging over the angular coordinate. This parameter quantifies the overall magnitude of membrane fluctuations along the cell contour, measured as angular roughness at one instant.

Second, each extracted h(θ) fluctuation function was subsequently transformed into Fourier space using a fast Fourier transform (FFT). The resulting fluctuation spectra, associated with lateral cortical tension (σ), display an inverse dependence on the equatorial wave vector $n_{q}$. Because the detected contours correspond to relatively large projected cell sizes, the analysis was confined to low-order (long-wavelength) deformation modes, typically around $n_{q}=3$. In this regime, membrane tension dominates the fluctuation spectrum ($q\ll \sqrt{\sigma /\kappa }$) as higher-order contributions are effectively buffered by the membrane–cortex area reservoirs. Under these conditions, an operational estimate of the effective lateral tension can be obtained from the relation $\sigma_{eff}∼k_{B}T/(2\pi n\;\langle |{\left.h_{n}\right|}^{2}\rangle ).$

Within this context, the ergodic flickering approach enables the analysis of shape fluctuations across hundreds of cells, yielding a robust ensemble-averaged estimate of lateral cortical tension by modeling the viscoelastic cortex as a thick membrane described by an effective Helfrich Hamiltonian. The mechanical contribution of the cortical cytoskeleton to membrane tension is incorporated as a spatially distributed force within the Helfrich continuum framework. In the ensemble-based (“ergodic”) approach, long-wavelength fluctuation statistics are estimated by treating the population of discocytic cells, acquired under matched conditions at a given time point, as an ensemble proxy for membrane states. This procedure assumes that, for the specific acquisition protocol used here (normocytic cells, in-focus equatorial contours, constant temperature and buffer, sub-hemolytic regime), ensemble variability across cells provides a practical proxy for temporal variability in single-cell flickering at the level of low-order modes. Importantly, this approximation is used here as a comparative estimator to quantify condition-dependent shifts under identical acquisition and processing, rather than as a route to absolute material parameters or to single-cell temporal inference.

### 2.8. High-Resolution & High-Speed Flickering Analysis

The second approach to investigate RBC dynamics involves the use of high spatial and temporal resolution videomicroscopy applied to a small cohort of cells for each experimental group.

RBC aliquots (40 µL) diluted 2:15 were loaded onto microscope slides using a silicone spacer chamber. Videos were acquired on a Nikon Eclipse TE2000 (Nikon Instruments Inc., Melville, NY, USA) inverted microscope using an Apo VC 100× oil objective with an additional 1.5× magnification, a Photron FASTCAM-SA3 camera (Photron Limited, Chiyoda-ku, Tokyo, Japan), and an additional 2.25× optical magnification, at a constant temperature of 37 °C. Videos were recorded at 2000 fps, 256 × 256 px (50 nm/px), for 3 s (6000 frames) and exported as uncompressed TIFF stacks. For each video, cell contours were extracted following the same procedure described in the previous section, including a denoising step, Otsu-based segmentation and posterior B-spline (polynomial degree 18) refinement and resampling (1024 angles per contour and time step). Contours are represented in polar form as $R\left(\theta ,t\right)=R_{0}+h(\theta ,t)$, relative to the instantaneous cell centroid [12]. To remove global translations and slow drift, the centroid was tracked frame-by-frame and the contour re-centered. Finally, the fluctuation field was defined as $h(\theta ,t)=R(\theta ,t)-\langle R(\theta ,t)\rangle_{t}$, where $\langle \cdot \rangle_{t}$ denotes time averaging over the full recording.

Mechanical parameters. For each of the 1024 time series of the cell contour, we computed the following descriptors:

***(A)***  *Signal volatility,* used as a measure of signal non-stationarity. It was defined as the variability of the fluctuation amplitude within a time series and estimated as the moving standard deviation of the signal amplitude, computed over 0.5 s windows with 0.25 s overlap [39].
***(B)***  *Effective local rigidity,* estimated under the assumption of thermal equilibrium as $k_{eff}=k_{B}T/{\left\langle h^{2}\right\rangle }_{t}$ where $\langle h^{2}\rangle_{t}$ denotes the time-averaged mean-square fluctuation amplitude (STD), which is equivalent to computing the mean squared displacements, $\left\langle h^{2}\left(\tau \right)\right\rangle$ MSD, value at τ→∞
***(C)***  *Einstein diffusivity,* extracted from the initial slope of the mean squared displacements $\left\langle h^{2}\left(\tau \right)\right\rangle =2\;D\;\tau$. Its inverse is related to the effective local viscosity of the cell membrane.
***(D)***  *Effective viscous friction,* obtained under an overdamped Langevin description of the local contour coordinate h(t), the Einstein relation links the short-time diffusivity to an effective friction coefficient γ as
  $$\begin{array}{ccc} D=\frac{k_{B}T}{\gamma }, & \Rightarrow & \gamma =\frac{k_{B}T}{D} \end{array}.$$
  here, D is obtained from the initial linear regime of the MSD. Thus, 1/D is proportional to the effective viscous dissipation, while γ provides an absolute friction proxy (in units of ${\mathrm{kg}\;\cdot \;s}^{-1}$) for the extracted contour coordinate.
***(E)***  *Characteristic relaxation frequency*, estimated as
  $$\omega_{c}\approx \frac{k_{eff}}{\gamma }=\frac{k_{eff}D}{k_{B}T},$$
  providing a single-cell proxy for the dominant relaxation timescale $\tau_{c}∼1/\omega_{c}$.

### 2.9. Statistical Analysis

Group comparisons were performed using nonparametric tests (Kruskal–Wallis for multi-group comparisons; Mann–Whitney U for planned pairwise tests). A two-sided significance threshold of α=0.05 was applied. Data are summarized as median and interquartile range (IQR). Mean ± SEM is not used for inferential comparisons in this single-donor pilot study. To minimize pseudo-replication, per-cell/per-video measurements were aggregated per donor prior to inferential testing whenever biological replication (multiple donors) was available. In the present study, statistics are therefore within-donor; *p*-values reflect cell-level sampling under a single biological replicate and should not be interpreted as population-level inference. Effects with 0.05≤p<0.1 are reported as trends only and are discussed cautiously without inferential claims. Because this is a single-donor pilot study, reported *p*-values reflect within-donor separability under matched acquisition/analysis and should not be interpreted as population-level inference.

All experiments were performed using erythrocytes from a single healthy donor and should therefore be considered a pilot study. The results support within-donor comparisons and methodological validation but do not allow population-level inference; generalization will require confirmation in multi-donor studies.

## 3. Results

### 3.1. Control of Erythrocyte Integrity: Hemolysis Assays

**Figure 3:**
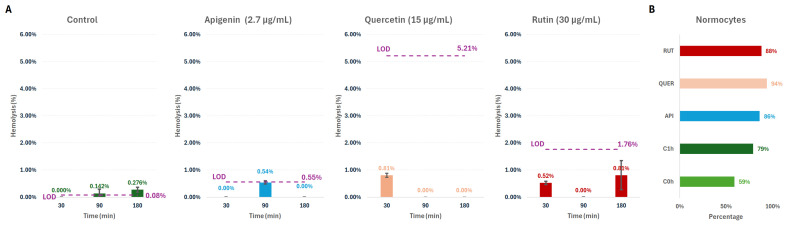
Hemolysis and morphotype quality control. (**A**) Time course of percent hemolysis in the supernatant, normalized to the dH_2_O positive control (100% hemolysis). Hemolysis was quantified for vehicle controls and for flavonoid-treated samples at multiple time points up to 3 h. The limit of detection (LOD) is reported for each effector and was derived from the method uncertainty (Appendix B.2). (**B**) Percentage of normocytes across conditions, estimated by computational morphotype classification from images. The normocyte fraction was computed as the ratio of cells meeting the normality criteria to the total number of cells detected in each field of view (see Figure 2). Sample size: 3 replicates per case.

To verify that the experimental conditions were compatible with subsequent biomechanical analyses, erythrocyte integrity was first assessed by hemolysis measurements under all incubation conditions. Hemolysis was quantified over a 3 h window, corresponding to the time frame used for morphometry and flickering experiments (Figure 3A). For each condition, we estimated a condition-specific limit of detection (LOD) by propagating the uncertainty associated with the absorbance measurements at the wavelengths used for hemoglobin quantification. Importantly, the LOD differed across effectors because the flavonoids exhibit residual spectral cross-talk, particularly due to emission tails approaching the lower-wavelength limit (~380 nm), which affects blank correction and increases the uncertainty budget (Appendix B.2).

In the vehicle control, hemolysis showed only a slight time-dependent increase, remaining negligible throughout the assay (<0.3%), consistent with basal incubation effects. For apigenin-, quercetin-, and rutin-treated samples, the estimated hemolysis values were similarly low (<0.5%) and, in all cases, fell below the corresponding LOD, indicating that the apparent fluctuations cannot be reliably interpreted as true changes in hemoglobin release. Taken together, these results confirm that the morphometric and flickering measurements performed at 0 h and 1 h were conducted within a sub-hemolytic regime, with no analytically detectable hemolytic effect or membrane disintegration under the selected imaging conditions.

Hemolysis measurements were complemented by an image-based morphotype analysis at 0 h and 1 h for controls and at 1 h for the flavonoid-treated samples (Figure 3B). The fraction of normocytes (biconcave discocytes) was computed for each condition; the remaining population consisted predominantly of echinocytic forms, with a residual representation of stomatocytes. The lowest normocyte fraction was observed at C0h (59%), consistent with mechanical and osmotic stress and ATP depletion during isolation [7], followed by a clear increment after 1 h incubation in PBS (79%). Under flavonoid exposure, the normocyte fraction increased further, reaching ~85–95% depending on the compound.

### 3.2. Morphometric Analysis of Erythrocytes

Bright-field images of erythrocytes were analyzed using a high-throughput morphometric pipeline to quantify changes in cell geometry and membrane-related mechanical proxies induced by incubation and flavonoid exposure. An initial morphometric screening indicated a predominantly discocytic population across all samples, with the remaining fraction consisting mainly of echinocytic and stomatocytic morphotypes (Figure 3B). These non-discocytic cells were excluded from further analysis because their 3D geometry frequently places portions of the membrane out of the focal plane, blurring the peripheral halo and compromising contour detection, thereby degrading the accuracy of downstream morphometric and mechanically inferred parameters. Beyond these practical limitations, non-discocytic morphologies are also expected to exhibit intrinsically different fluctuation physics: changes in shape are accompanied by substantial shifts in effective mechanical state (e.g., excess-area availability, tension and stiffness) and in the fluctuation spectrum, as shown for discocyte–echinocyte–spherocyte transitions [7,11]. In particular, stomatocytic configurations are typically dominated by altered curvature/tension constraints, while echinocytic phenotypes involve spicule-related heterogeneities and membrane–cytoskeleton remodeling that can strongly bias mode decomposition and relaxation dynamics. Downstream analyses were therefore restricted to discocytic (normocytic) cells to ensure robust contour-based quantification and meaningful cross-condition comparisons of flickering-derived phenotypes.

Six morphomechanical parameters were considered: mean equivalent radius, elongation, circularity, elongation, fluctuation amplitude, shape factor, and effective tension. In all analyses, both the baseline vehicle control measured immediately after extraction (C0h) and the incubated vehicle control measured after 1 h at 37 °C (C1h) were included to contextualize the post-isolation state.

**Figure 4:**
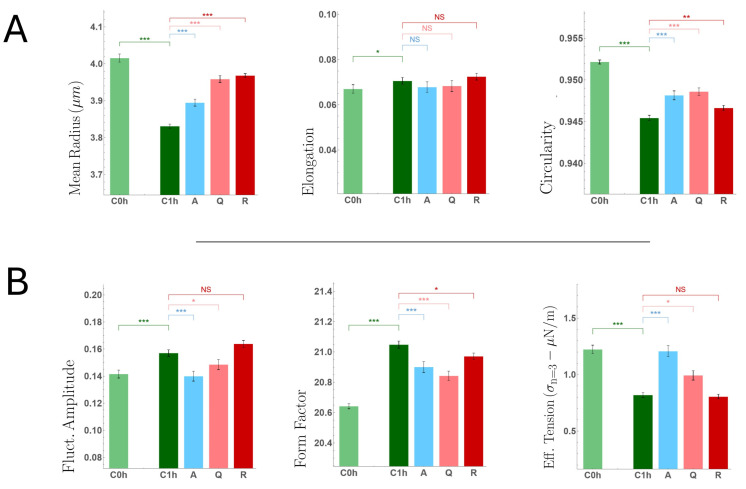
RBC morphometric analysis. Six morphology-related parameters were extracted from bright-field images for controls (0 h and 1 h) and flavonoid-treated samples (1 h): (**A**) equivalent mean radius, defined as the radius of a circle with the same area; elongation, E=a/b, where a and b are the major and minor semi-axes of the inertia-equivalent ellipse; circularity, $C=4\pi A/P^{2}$; (**B**) Fluctuation amplitude, estimated as the radial contour variability, reflecting angular roughness; form factor, $F=P^{2}/A$; and effective tension inferred from low-mode spectral analysis. Box plots summarize cell-level distributions (median and IQR). Statistical comparisons were performed using the Mann–Whitney test. Statistical significance: * *p* < 0.05, ** *p* < 0.01, *** *p* < 0.001. Values with 0.05 ≤ *p* < 0.1 are reported as trends and explicitly indicated in the text, but are not interpreted as statistically significant (NS). Because all measurements are from a single donor, statistical tests here quantify within-donor separability across conditions under matched acquisition/analysis, and should not be interpreted as population-level inference. Sample size: 300–400 cells per case.

Representative images showed predominantly normocytic morphologies across all conditions, with no evidence of shape transitions or widespread echinocytic or spherocytic forms (Figure 4). Neither circularity nor elongation shows noticeable modifications under incubation with vehicle (PBS+ + DMSO). However, quantitative analysis revealed a clear and statistically significant effect of the incubation step when comparing C0h and C1h controls (see Table 2). After 1 h of incubation, all analyzed parameters exhibited systematic shifts consistent with metabolic reactivation following the osmotic and mechanical stress associated with erythrocyte purification. Specifically, morphometric descriptors indicated a marked reduction in cell size and circularity, while the global cell shape, as quantified by elongation, remained essentially unchanged. In parallel, mechanical-related parameters showed an increase in membrane fluctuation amplitude and shape factor, accompanied by a significant decrease in effective tension [6,9,40]. Taken together, these trends point to a progressive incubation-dependent relaxation from post-isolation perturbation, associated with increased membrane dynamical activity and mechanical compliance from post-isolation perturbation after sample preparation.

Comparison of flavonoid-treated samples with the C1h incubated control revealed compound-dependent morphomechanical signatures. No significant changes were observed in elongation for any treatment, indicating that global cell geometry was preserved. In contrast, all three flavonoids induced a measurable increase in cell size, accompanied by a slight but consistent increase in circularity. This effect was more pronounced for apigenin and quercetin (both aglycones), whereas rutin-treated cells showed minimal changes relative to the vehicle.

Analysis of membrane fluctuation amplitude further highlighted distinct behaviors among the compounds. Rutin-treated erythrocytes exhibited fluctuation amplitudes comparable to the vehicle control, indicating no appreciable modification of membrane dynamics. In contrast, treatment with quercetin and apigenin resulted in a significant reduction in the mean fluctuation amplitude. This trend was mirrored by corresponding changes in the shape factor, which decreased in aglycone-treated samples but remained unchanged in the presence of rutin.

Finally, estimation of the effective mechanical tension revealed a clear divergence between aglycones and the glycosylated flavonoid. Apigenin- and quercetin-treated cells displayed a significant increase in effective tension relative to vehicle control, whereas no detectable change was observed for rutin-treated erythrocytes. Consistent with prior validations, the effective tensions extracted from ensemble-averaged static spectra fall within the range reported for time-resolved flickering analyses of healthy erythrocytes [9,39], supporting their interpretation as comparative mechanical descriptors rather than methodological artifacts. These results indicate that, despite the absence of major geometric alterations, aglycones induce a mechanically stiffer membrane phenotype, while rutin preserves morphomechanical properties close to the control state.

### 3.3. Flickering Analysis: Dynamic and Mechanical Signatures

High spatiotemporal resolution microscopy was used to perform a detailed analysis of local membrane fluctuations in erythrocytes under the different experimental conditions. For each group, fluctuations were analyzed in ten individual cells. In the case of apigenin-treated samples, five recordings were excluded due to insufficient image quality or contour-tracking reliability, resulting in five cells included in the final analysis. All reported results are therefore based on high-quality single-cell time series suitable for quantitative flickering analysis.

Rheological descriptors derived from fluctuation dynamics revealed consistent and compound-dependent trends. Effective rigidity and the inverse of Einstein diffusivity (used here as a proxy for viscous dissipation) displayed closely aligned behaviors across conditions. Both apigenin- and quercetin-treated erythrocytes exhibited a clear increase in effective rigidity accompanied by an increase in viscous friction, indicative of enhanced membrane viscoelasticity. In contrast, rutin-treated cells did not show statistically significant deviations from vehicle control in either rheological parameter, remaining within the control variability range.

To disentangle the respective roles of elastic stiffening and viscous dissipation, we analyzed the coupling between effective rigidity and the characteristic relaxation frequency at the single-cell level. For each cell, we estimated the relaxation frequency as $\omega_{c}∼k_{eff}/\gamma$, where the effective friction γ was obtained from the short-time Einstein diffusivity via $\gamma =k_{B}T/D$. We then plotted $\omega_{c}$ against $k_{eff}$ to visualize how changes in stiffness and dissipation jointly reshape fluctuation timescales across conditions. This representation showed that apigenin- and quercetin-treated cells shift toward higher $\omega_{c}$ as $k_{eff}$ increases, indicating faster relaxation despite concomitantly increased friction. Because the characteristic relaxation frequency scales as $\omega_{c}∼k_{eff}/\gamma$, an increase in $\omega_{c}$ reflects a dominant stiffening effect that outweighs the concomitant increase in viscous dissipation, rather than a reduction in friction. Consequently, the different experimental groups populate distinct regions of the $\left(k_{eff},\;\omega_{c}\right)$ plane, following an approximately linear trend that separates aglycone-treated cells from controls and rutin-treated cells.

This combined representation defines a characteristic mechanical space in which flavonoid-treated erythrocytes are displaced relative to controls, consistent with a shift toward a mechanically more constrained dynamical regime, characterized by increased effective rigidity and shorter relaxation timescales. In contrast, rutin-treated cells clustered near the vehicle control, reinforcing the absence of a significant mechanical remodeling under this condition. All inferred quantities are reported as effective descriptors within the adopted fluctuation-inference model and should not be interpreted as absolute bilayer or cytoskeletal material constants. We use them comparatively to quantify condition-dependent shifts under matched acquisition and analysis.

Finally, signal volatility was analyzed as a measure of flickering non-stationarity. Quercetin-treated erythrocytes displayed a marked reduction in signal volatility compared with vehicle controls, indicating a stabilization of fluctuation amplitudes over time. This effect was not statistically significant for apigenin- or rutin-treated samples, which exhibited volatility levels comparable to the control group. Together, these observations suggest that quercetin uniquely reduces temporal heterogeneity in membrane dynamics, whereas apigenin primarily affects average mechanical properties without substantially altering signal non-stationarity.

### 3.4. Integrative Phenotypic Analysis

**Figure 5:**
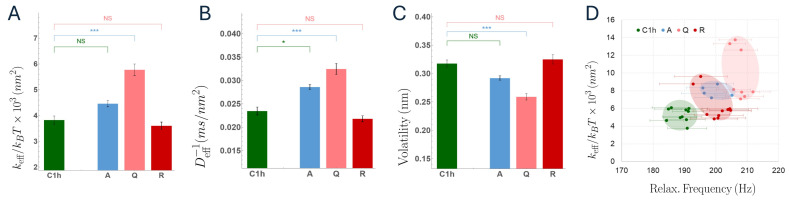
RBC flickering results. Three main parameters were obtained from the analysis of local membrane fluctuations. (**A**) effective rigidity, reflecting local resistance to linear membrane deformation (**B**) inverse diffusivity, from which an effective friction coefficient was obtained via $\gamma =k_{B}T/D$. (**C**) volatility, used as an activity-related marker (non-stationarity). Box plots summarize cell-level distributions (median and IQR). (**D**) Cross-parameter analysis of local rigidity as a function of the relaxation frequency. Statistical significance: * *p* < 0.05, *** *p* < 0.001. Values with 0.05 ≤ *p* < 0.1 are reported as trends and explicitly indicated in the text, but are not interpreted as statistically significant (NS). Sample size: 20 cells, 60 videos per case.

To explore the relationship between static geometry and dynamic membrane behavior, morphometric and flickering-derived parameters were analysed jointly. Scatter plots revealed partial correlations between selected dynamic descriptors (e.g., characteristic relaxation frequency or effective rigidity) (Figure 5C) and morphometric measures such as equivalent radius or circularity. These correlations were moderate and condition-dependent, indicating that morphological and dynamic readouts capture overlapping but non-redundant aspects of the erythrocyte membrane state. Importantly, the combined analysis highlighted that flavonoid-induced phenotypic signatures cannot be fully described by morphology or flickering alone; instead, their integration provides a more sensitive and discriminative characterization.

The static (ensemble-based) and dynamic (time-resolved) analyses probe complementary projections of the same underlying membrane–cortex mechanics. It is important to note that effective (ensemble) tension and effective rigidity are distinct mechanical readouts and do not imply one another a priori. While the ergodic static spectra emphasize long-wavelength, population-averaged constraints dominated by effective tension, the time-resolved analysis captures local viscoelastic response and dissipation at shorter timescales. Under the assumption of a shared effective mechanical state, consistent compound-dependent trends across these two readouts (such as concurrent increases in effective tension, rigidity, and viscous friction) support a unified interpretation in terms of membrane remodeling rather than independent or contradictory effects.

Taken together, the results demonstrate that, under sub-hemolytic conditions, structurally distinct flavonoids induce reproducible and differentiable phenotypic signatures in erythrocyte morphology and membrane dynamics. Within the limitations of a single-donor pilot study, these findings support the feasibility and sensitivity of the combined morphometry–flickering approach as a biophysical phenotyping framework.

## 4. Discussion

### 4.1. Mechanical Phenotyping of Flavonoid-Treated RBCs by Computational Microscopy

This work shows that a computational microscopy workflow that combines single-cell morphometry with flickering spectroscopy can resolve compound-specific mechanical phenotypes of RBCs under flavonoid exposure. Flickering is a sensitive readout because it integrates thermal fluctuations, bending resistance, membrane tension, cytoskeletal elasticity, and metabolism-dependent activity, so it can respond to perturbations that remain morphologically subtle [6,7,8]. Consistent with this view, our measurements reveal that dynamic signatures can shift even when mean shape remains largely discocytic and membrane integrity is preserved, supporting the use of flickering as a systems-level marker of effective envelope mechanics [8,10,40].

### 4.2. Non-Redundancy of Morphometry and Flickering

A key implication is that morphology and flickering report complementary aspects of the membrane system. Morphometry reflects time-averaged geometry and constraints, whereas flickering encodes effective mechanical response across spatiotemporal scales. Previous work has established that fluctuation spectra and their spatial correlations can be used to infer effective descriptors and to interrogate the contribution of active processes [8,10,40]. Our results reinforce that a compound can alter fluctuation dynamics without producing an obvious mean shape transition, which motivates joint phenotyping rather than reliance on single endpoint readouts.

### 4.3. Structure-Dependent Membrane Interactions Across Flavonoids

The differences observed among quercetin, apigenin, and rutin are consistent with established determinants of flavonoid membrane affinity and insertion. Flavonoid interactions depend on hydroxylation pattern, molecular planarity and conjugation, and glycosylation, which increases polarity and typically reduces bilayer partitioning [14,15,16]. Accordingly, rutin should not be expected to behave as a simple equivalent of quercetin at the membrane interface, and scaffold level differences between apigenin and quercetin can further tune membrane effects [17,18,20]. Aglycones are expected to accumulate at the lipid–water interface, where phenolic OH groups can hydrogen-bond with phospholipid headgroups while the aromatic scaffold partially inserts into the upper acyl-chain region [15]. Such interfacial insertion can increase local lipid packing/order, providing a plausible basis for the concurrent shifts in effective rigidity and viscous damping observed here. In contrast, glycosylation (rutin) increases polarity and hydration, favoring a more superficial localization with weaker perturbation of chain packing and therefore a smaller mechanical phenotype.

In this framework, compound-specific flickering phenotypes most naturally reflect differences in partition depth and lipid ordering rather than generic antioxidant capacity. Rutin shows a weaker mechanical phenotype consistent with reduced bilayer partitioning expected for glycosylation; antioxidant effects are not directly assayed here. More generally, because erythrocyte mechanics reflect a bilayer–cytoskeleton composite, insertion-driven changes in bilayer packing can also modulate bilayer–skeleton friction, naturally linking rigidity and viscous damping within a unified membrane-coupling picture.

### 4.4. Cholesterol Context and the Escin Comparison as a Mechanistic Benchmark

Membrane composition is central to interpretation because cholesterol regulates bilayer order, lateral organization, and mechanical coupling to the cytoskeleton, and it can modulate flavonoid effects in erythrocytes under oxidative stress [23,24]. Because cholesterol tunes headgroup spacing and acyl-chain order in the same depth range targeted by interfacial small-molecule insertion, cholesterol-dependent partitioning provides a direct mechanistic route to compound- and protocol-dependent variability [15]. This cholesterol dependence connects directly to previous work on beta escin, which forms compact complexes with membrane cholesterol and acts as a membrane stiffener in model bilayers [9]. Cholesterol tunes headgroup spacing and chain order in the same depth range targeted by aglycones, so cholesterol-dependent partitioning offers a mechanistic explanation for reported variability across studies. Importantly for the present work, escin provides a controlled benchmark to contrast passive bilayer stiffening with mechanically adaptive responses in living RBCs [25,26]. In living RBCs, escin-treated cells maintain biologically active flickering within tolerability limits, consistent with homeostatic mechanical adaptability, whereas in passive giant vesicles, escin stiffens the bilayer and suppresses thermal fluctuation power without adaptive regulation [27]. This contrast strengthens the interpretation that changes in flickering under membrane active compounds can reflect regulated coupling between the bilayer and cytoskeleton, not only passive mechanical constraints. From a chemical standpoint, escin is not a flavonoid, but it does share an amphiphilic architecture that includes a membrane-active aglycone core and glycosidic substituents that tune partitioning and membrane organization. The same conceptual motif appears in flavonoid chemistry, where glycosylation differentiates rutin from aglycones such as quercetin and apigenin, often shifting membrane affinity and therefore mechanical impact [14,15,16]. Thus, escin offers a useful mechanistic benchmark for interpreting how aglycone-driven membrane activity and sugar-mediated polarity modulation shape compound-specific mechanical phenotypes in erythrocytes.

### 4.5. Interpretation of Extracted Mechanical Descriptors

The parameters extracted from contour fluctuations should be interpreted as effective descriptors of projected dynamics within the adopted model assumptions, rather than absolute material constants of the lipid bilayer or cytoskeleton. This caveat is standard in fluctuation-based inference and is particularly relevant for comparisons across compounds, where the primary goal is to quantify relative shifts under matched acquisition and analysis conditions [8,10]. Similarly, any phenomenological measures of fluctuation activity should be viewed as comparative markers of dynamic state unless independently linked to a molecular mechanism. The mechanical inference strategy adopted here, linking fluctuation amplitudes and spectral scaling to effective mechanical descriptors, has been independently validated in related fluctuation-inference settings by García-García et al. (Nature Communications, 2025) [39], supporting the use of this framework for comparative discrimination under controlled perturbations. Although applied here to erythrocyte membranes, this prior validation supports the general reliability of the inference framework when used comparatively under homogeneous acquisition and analysis conditions. Thus, the present trends are best interpreted as compound-dependent shifts in effective mechanical state rather than analysis-dependent artifacts. Since the static spectra and the time-resolved analysis emphasize different physical regimes, changes in effective tension and rigidity can be decoupled a priori: tension mainly constrains long-wavelength fluctuations, whereas rigidity and friction shape short-scale response and relaxation kinetics. Accordingly, “more tension” does not imply “more rigidity” a priori, and the two readouts should not be interpreted interchangeably. Hence, the observation of concurrent shifts in tension, rigidity, and friction should be read as a coherent multi-regime signature rather than a tautological equivalence between terms. Taken together, the simultaneous increase in effective tension (from ensemble-averaged static spectra) and the increase in effective rigidity accompanied by higher dissipation (from time-resolved analysis) support a coherent interpretation: aglycone flavonoids shift the membrane–cortex system toward a mechanically more constrained dynamical regime. This shift is consistent with increased membrane ordering and/or altered bilayer–cytoskeleton coupling, which can elevate both elastic resistance and viscous damping. We use the term “mechanically cooled” only as a phenomenological descriptor of reduced fluctuation timescales and a more strongly damped regime; it should not be interpreted as a literal decrease in an effective thermodynamic temperature. Rather, it reflects a compound-dependent rebalancing between stiffness and dissipation within an active membrane–cortex system. Because RBC mechanics reflect a bilayer–cytoskeleton composite, insertion-driven changes in bilayer packing can also shift bilayer–skeleton friction, naturally linking stiffness and effective friction changes. These interpretations are consistent with established models of flavonoid–membrane interactions, where partitioning depth and lipid composition critically determine mechanical and biological outcomes [15]. Mechanistically, the present phenotypes are most consistent with a combination of partition-depth–dependent lipid ordering and altered bilayer–cytoskeleton friction; these contributions can be directly tested in future work via cholesterol depletion/loading, membrane-order readouts (e.g., Laurdan generalized polarization), and ATP/metabolic modulation.

### 4.6. Limitations, Mechanistic Anchoring, and Future Directions

The ergodic estimator is expected to be most reliable for homogeneous morphotypes and for long-wavelength modes where projection and contour extraction are robust. It may be biased by shape heterogeneity, out-of-plane membrane segments, or strong non-stationarity in active processes. For this reason, we restrict the ergodic analysis to discocytic (normocytic) cells meeting strict segmentation and morphometry criteria, and we interpret the resulting effective tension strictly at the phenotype level (within-donor, within-protocol comparisons). A dedicated dataset-specific cross-validation between ensemble and time-resolved estimators is beyond the scope of this proof-of-concept study and is a planned extension for future pipeline releases.

The current study is a within-donor pilot design and therefore supports robust condition comparisons and methodological validation, but it does not support population-level inference. Accordingly, all statistical tests reported here quantify within-donor separability across experimental conditions under matched acquisition and analysis workflows, and should not be interpreted as evidence for inter-individual variability or generalizable population effects. Future work should extend the analysis to multiple donors, include controlled modulation of membrane cholesterol, and establish dose-response curves for each flavonoid. A natural next step is to place flavonoids and escin within the same phenotyping space to test whether compounds cluster according to shared amphiphilic logic, cholesterol sensitivity, and their ability to elicit adaptive versus passive fluctuation responses, as suggested by the escin comparison between living cells and passive vesicles [26,27]. Such extensions would further position flickering-based computational microscopy as a scalable tool for membrane biophysics and for screening membrane-active molecules through their non-equilibrium mechanical fingerprints. Direct mechanistic anchoring at the membrane-chemistry level remains an important next step.

Future work will combine flickering-based mechanical phenotyping using the “ergodic” method with independent readouts of membrane organization and partitioning, including controlled cholesterol depletion/loading, membrane order probes (e.g., Laurdan GP and Flipper-TR), and dose–response analyses for each flavonoid. Integrating these approaches will enable quantitative mapping between physicochemical partitioning depth, lipid ordering, and non-equilibrium mechanical response, and will clarify how structurally distinct amphiphiles position erythrocytes along a continuum between adaptive and passive membrane remodeling.

## 5. Conclusions

In this work, we introduce a computational microscopy framework that integrates erythrocyte morphometry and membrane flickering to phenotype membrane mechanics under flavonoid exposure. Using quercetin, apigenin, and rutin as model compounds, we show that within this single-donor framework, structurally distinct flavonoids induce reproducible and distinguishable single-cell mechanical signatures under sub-hemolytic conditions. Aglycone flavonoids shift erythrocytes toward a mechanically more constrained dynamical regime, characterized by increased rigidity and faster relaxation times, whereas the glycosylated flavonoid rutin produces weaker effects. Although absolute mechanical parameters should be interpreted comparatively, the consistency of trends across cells demonstrates the sensitivity of the approach. This single-donor study highlights the value of combining static and dynamic readouts to resolve membrane-active perturbations and establish a foundation for future multi-donor and mechanistically anchored studies.

## Figures and Tables

**Table 1:** Physicochemical properties of the selected flavonoids. LogP, water solubility, and topological polar surface area (TPSA) values were compiled from established physicochemical databases and literature sources [14,15,16,31,32], as detailed in Appendix A. LogP corresponds to XlogP3-AA where available; solubility values correspond to aqueous solubility entries reported in PubChem (compound record), and TPSA corresponds to the computed topological polar surface area.

	Apigenin	Quercetin	Rutin
Flavonoid Family	Flavone (aglycone)	Flavonol (aglycone)	Glycosylated flavonol
Molar mass	270	302	610
Log P (hydrophobicity)	2.84	1.81	−1.97
Water solubility	1.35 µg/mL	2.15 µg/mL	125 µg/mL
TPSA	91 Å^2^	131 Å^2^	269 Å^2^
Interaction with RBC *	Interfacial compound with biphasic behavior	Interaction with the bilayer and Hb/Fe compartment. Clear cholesterol dependence	Higher polarity; reduced bilayer partitioning expected; likely more superficial localization (hypothesized)

* Expected behavior based on bibliography review. See Appendix A.

**Table 2:** Statistical analysis of morphometric parameters across experimental groups. Pairwise comparisons of the distributions of morphometric parameters among all experimental groups for which morphometric measurements were performed (as shown in Figure 4), including both controls (C0h and C1h) and effector-treated samples. Reported values are *p*-values from Mann–Whitney U tests assessing differences in group means/distributions. Significance levels: * *p* < 0.05, ** *p* < 0.01, *** *p* < 0.001. Values with 0.05 ≤ *p* < 0.1 are reported as trends and explicitly noted in the text, but are not interpreted as statistically significant (NS).

Property	COh vs. C1h	COh vs. A	COh vs. Q	COh vs. R	C1h vs. A	C1h vs. Q	C1h vs. R
Mean Radius	***	***	***	***	***	***	***
Elongation	*	NS	NS	*	NS	NS	NS
Circularity	***	***	***	***	***	***	**
Flut. Amplitude	***	NS	NS	***	***	*	NS
Form factor	***	***	***	***	***	***	*
Effective tension	***	NS	NS	***	***	*	NS

## Data Availability

The data presented in this study are available from the corresponding authors upon reasonable request.

## Appendix A

**Brief literature notes on the interaction of apigenin, rutin, and quercetin with red blood cells (RBCs).**

Physicochemical descriptors (logP, water solubility, TPSA) reported in Table 1 were obtained from curated databases (PubChem, DrugBank) and cross-checked against review literature on flavonoid–membrane interactions [14,15,16]. Values on interaction parameters are reported to support relative comparisons rather than absolute quantitative prediction of membrane partitioning.

This annex provides a brief, evidence-based rationale for the distinct “interaction modes” assigned to apigenin, quercetin, and rutin in the main manuscript table. In RBCs, these compounds may affect membrane behavior either by associating with the lipid–water interface (thereby influencing vulnerability to lipid peroxidation) and/or through indirect redox pathways that preserve or perturb membrane integrity via effects on hemoglobin oxidation, labile iron chemistry, and protein/cytoskeletal damage.

**Rutin** is usually described as an interfacial antioxidant “shield” because its glycosylated structure is relatively hydrophilic and bulky, making deep penetration into the hydrophobic bilayer core less favorable. The RBC literature most consistently supports rutin as reducing oxidative hemolysis and lipid peroxidation, consistent with activity near the membrane surface/interfacial region. Despite clear antioxidant protection, there is little direct evidence that rutin substantially embeds in the inner hydrophobic bilayer or drives primary mechanical remodeling.

**Quercetin** is described as a dual interface and interior interaction because, as an aglycone, it can partition into membrane interfaces and is also taken up by RBCs, where it associates with hemoglobin and can influence Hb/Fe-linked redox chemistry. This dual-compartment behavior helps explain why quercetin often shows stronger protection than rutin in oxidative stress models and why its efficacy is sensitive to membrane cholesterol, which modulates interfacial packing and partitioning. In this framework, quercetin’s higher potency reflects combined interfacial/bilayer protection against lipid oxidation and intracellular modulation of hemoglobin/iron-driven amplification of membrane injury.

**Apigenin** is described as interfacial with biphasic behavior because RBC studies support two dose–time regimes. Under acute oxidative stress, apigenin can act as a cytoprotective antioxidant, reducing lipid peroxidation and preserving membrane/cytoskeletal components. In contrast, at sustained micromolar concentrations and prolonged incubation, apigenin has been reported to trigger eryptosis markers (Ca^2+^ rise, shrinkage, phosphatidylserine exposure) without necessarily causing immediate hemolysis; thus, the “risk of triggering eryptosis” refers to a distinct phenotype involving loss of lipid asymmetry and pro-clearance/procoagulant signaling outside the protective dose–time window.

### Appendix A.1. Rutin: Interfacial Antioxidant Shield with Limited Insertion

Suppression of oxidative hemolysis and lipid peroxidation at the cell/membrane interface. In photosensitized hemolysis of human RBCs (hematoporphyrin), both rutin and quercetin suppressed hemolysis accompanied by inhibition of lipid peroxidation; both flavonols were consumed (photooxidized), consistent with a scavenging role close to the initiating oxidative chemistry [41].

Protection against tert-butyl hydroperoxide (tBHP)-induced oxidative injury with cholesterol sensitivity. In RBCs exposed to oxidative stress, rutin reduces damage metrics (lipid peroxidation/ROS-related endpoints) but typically with lower potency than quercetin; importantly, protection depends on membrane cholesterol content [23,24].

Preservation of redox-related readouts (GSH/Heinz bodies) under oxidative challenge. Under tBHP, rutin contributes to attenuation of oxidative stress markers in human RBCs, although specific endpoints may differ vs quercetin depending on the protocol [42].

Antioxidant protection against radical-driven hemolysis. In radical-induced hemolysis paradigms, rutin is consistently reported as anti-hemolytic, supporting a dominant antioxidant mode [43].

Mechanistic interpretation. Rutin is a glycosylated flavonol (rutinoside), which increases hydrophilicity and steric bulk, making deep penetration into the hydrophobic core less favorable compared with aglycones. The RBC literature mainly supports rutin as a surface/interfacial antioxidant that attenuates oxidative membrane injury (hemolysis and lipid peroxidation), with limited direct evidence for deep insertion or primary mechanical remodeling.

Limits of evidence. Direct localization depth in RBC membranes (e.g., multi-depth ESR spin probes, Laurdan GP stratification) is rarely reported specifically for rutin in intact RBCs. Thus, “limited insertion” is supported primarily by (i) structure-based plausibility and (ii) the predominance of antioxidant/hemolysis outcomes rather than direct bilayer-depth measurements.

### Appendix A.2. Quercetin: Interface & Interior Interaction with Higher Potency and Cholesterol Dependence

Rapid uptake and near-quantitative accumulation driven by hemoglobin binding (interior compartment). Human RBCs take up quercetin rapidly via passive diffusion, with accumulation driven by binding to hemoglobin; in hemoglobin-free resealed ghosts, quercetin accumulates exclusively in the membrane fraction [44].

Membrane interfacial interaction evidenced by EPR readouts. EPR-based work supports quercetin incorporation affecting the interfacial environment of RBC membranes [20].

Strong protection against oxidative injury with explicit cholesterol dependence. Quercetin provides stronger protection than rutin in multiple oxidative endpoints (lipid peroxidation/ROS, integrity/viability), and both its protective efficacy and the oxidative phenotype are modulated by membrane cholesterol [23,24].

Iron-related mechanisms and protection of membrane proteins under oxidative stress. Quercetin can protect against oxidative membrane damage in ways consistent with iron chelation and prevention of oxidative alterations of membrane proteins [19].

Functional coupling to RBC membrane proteins (band 3) and transport. Flavonoids (including quercetin) can modulate anion exchange through band 3, linking membrane interaction to functional readouts [45].

Mechanistic interpretation. Quercetin combines (i) interfacial membrane partitioning (supported by EPR/protection patterns and strong cholesterol sensitivity) with (ii) intracellular accumulation via Hb binding, and potentially iron-related redox chemistry. This dual-compartment behavior provides a coherent explanation for its commonly observed higher potency vs rutin against oxidative injury, as well as the pronounced dependence of outcomes on membrane cholesterol (which controls interfacial packing/partition energetics) and the ability to protect both lipid and protein components under oxidant stress.

Limits of evidence. While uptake/Hb binding is well supported, the quantitative partition coefficients and depth distribution in native RBC membranes remain incompletely mapped across studies and protocols (vehicle, time, cholesterol manipulation). Mechanical readouts (ektacytometry, AFM) are also less common than oxidative endpoints.

### Appendix A.3. Apigenin: Interfacial Interaction with Biphasic Behavior (Protection vs. Eryptosis)

Protection against H2O2-induced oxidative damage. Apigenin reduces hemolysis and lipid peroxidation markers, preserves sulfhydryl groups in membrane proteins, and improves morphology/ultrastructure of the membrane skeleton under oxidative challenge [29].

Inhibition of oxidative processes in RBCs with micromolar potency. In a tBHP model, apigenin inhibits lipid peroxidation in RBC-related assays with a micromolar IC50 reported in the study [30].

Apigenin-induced eryptosis (suicidal RBC death). Apigenin increases cytosolic Ca^2+^, promotes cell shrinkage and phosphatidylserine exposure, and is associated with ceramide formation and ATP depletion under prolonged incubation, without necessarily causing overt hemolysis [28].

Mechanistic interpretation. Apigenin is an aglycone flavone with physicochemical features consistent with interfacial partitioning and partial insertion (mechanistic support from model membranes), which aligns with its ability to attenuate oxidative membrane injury. However, at sustained micromolar exposures and long incubation times, apigenin can activate hallmark pathways of eryptosis (Ca^2+^-linked signaling, PS exposure, shrinkage), representing a distinct regime with potential implications for RBC clearance and procoagulant surface changes.

Limits and physiological relevance. Concentrations and incubation times required to trigger eryptosis in vitro may exceed typical dietary plasma levels; nevertheless, the observation is important for defining safe experimental windows and for interpreting long incubations [46]. Direct depth-resolved measurements of apigenin within native RBC membranes are limited; model-membrane spectroscopy supports interfacial interaction but should not be over-extrapolated [20].

## Appendix B

### Appendix B.1. Hemolysis Quantification and Harboe Method with Allen Correction

For hemolysis percentage quantification, the Harboe method was used. It started by determining the optical density of the samples at 415 nm (oxyhemoglobin), and corrections at 380 nm (plasmatic impurities) and 450 nm (bilirubin-albumin complexes) [34,35,36]. Before any calculation, the optical density values of the control samples were adjusted by detracting the measurement at time zero, while the effector samples were adjusted using the values measured in the vehicle blanks. Corrected values were then used as input for quantification of hemoglobin concentration in the Harboe (with Allen correction) equation as

$$C_{Hb}=df\cdot \;\frac{167.2\cdot A_{415nm}-83.6\cdot A_{380nm}-83.6\cdot A_{450nm}}{1000}$$

being df de dilution factor. The percentage of hemolysis was calculated by comparison with the positive control as

$$\%\;Hemolysis=\frac{{C_{Hb}}_{sample}}{{C_{Hb}}_{dH_{2}O}}$$

### Appendix B.2. Limit of Detection and Uncertainty Calculation

To determine the uncertainty in the estimation of the quantified hemoglobin concentration, we use Gauss’s law of propagation of errors, according to which

$$u_{C_{Hb}}=\sqrt{{\left(u_{A_{415}}\frac{\partial C_{Hb}}{\partial A_{415}}\right)}^{2}+{\left(u_{A_{380}}\frac{\partial C_{Hb}}{\partial A_{380}}\right)}^{2}+{\left(u_{A_{450}}\frac{\partial C_{Hb}}{\partial A_{450}}\right)}^{2}}$$

Derivatives are constant

$$\frac{\partial C_{Hb}}{\partial A_{415}}=\frac{167.2}{1000}\;\frac{\partial C_{Hb}}{\partial A_{380}}=-\frac{38.6}{1000}\;\frac{\partial C_{Hb}}{\partial A_{450}}=-\frac{38.6}{1000}$$

Assuming an uncertainty of ±0.005 in the absorbance estimate (provided by the manufacturer), independent of the wavelength, then the uncertainty in the determination of the hemoglobin concentration is

$$u_{Hb}=\frac{df}{1000}\sqrt{{\left(u_{A_{415}}\cdot 167\right)}^{2}+{\left(u_{A_{380}}83.6\right)}^{2}+{\left(u_{A_{450}}83.6\right)}^{2}}$$

The uncertainty at each wavelength is determined from the standard error of the measurement (triplicates) of the effector cell-free samples.

As previously stated, the percentage of hemolysis can be estimated through the ratio between the amount determined for a test and the amount estimated for the positive control

$$R=\frac{{C_{Hb}}_{sample}}{{C_{Hb}}_{dH_{2}O}}$$

Similarly, using the propagation of uncertainties, we establish that the uncertainty in the estimation of this ratio is

$$u_{R}=\sqrt{{\left(u_{Hb\;}\frac{\partial R}{\partial C_{Hb}}\right)}^{2}+{\left(u_{Hb-dH_{2}O\;}\frac{\partial R}{\partial {C_{Hb}}_{dH_{2}O}}\right)}^{2}+2\;\frac{\partial R}{{C_{Hb}}_{dH_{2}O}}\;\frac{\partial R}{\partial C_{Hb}}Cov\;({C_{Hb}}_{dH_{2}O},C_{Hb})}$$

Derivatives then are

$$\frac{\partial R}{\partial C_{Hb}}=\frac{1}{{C_{Hb}}_{dH_{2}O}}\;\frac{\partial R}{\partial {C_{Hb}}_{dH_{2}O}}=-\frac{C_{Hb}}{{\left({C_{Hb}}_{dH_{2}O}\right)}^{2}}=-\frac{R}{{C_{Hb}}_{dH_{2}O}}$$

Furthermore, assuming equivalent uncertainty for both estimates $u_{C_{Hb}}=u_{C_{Hb-sample}}$, as well as independence in the determination of both magnitudes (zero covariance), the uncertainty is

$$u_{R}=R\;u_{C_{Hb}}\sqrt{\frac{1}{C_{Hb\;}^{2}}+\frac{1}{{C_{Hb}}_{dH_{2}O}}}$$

At the limit of low *Hb* concentrations $\left(C_{Hb}\ll {C_{Hb}}_{dH_{2}O}\right)$, the uncertainty in the measurement of the ratio becomes

$$u_{R}\approx \frac{u_{C_{sample}}}{{C_{Hb}}_{dH_{2}O}}$$
